# Application of Discrete Choice Experiment in Health Care: A Bibliometric Analysis

**DOI:** 10.3389/fpubh.2021.673698

**Published:** 2021-06-04

**Authors:** Yue Wang, Zhangyi Wang, Zhao Wang, Xuechun Li, Xiaoli Pang, Shuling Wang

**Affiliations:** ^1^The School of Graduate, Tianjin University of Traditional Chinese Medicine, Tianjin, China; ^2^The School of Nursing, Tianjin University of Traditional Chinese Medicine, Tianjin, China; ^3^The Second Affiliated Hospital of Tianjin University of Traditional Chinese Medicine, Tianjin, China

**Keywords:** discrete choice experiment, conjoint analysis, health care, survival, bibliometric analysis

## Abstract

**Background:** Discrete choice experiment (DCE) as a tool that can measure medical stakeholders' preferences especially patients recently has been increasingly applied in health care.

**Objective:** The aim of this study was to examine the hotspots and trends of the application of DCE in health care and to provide reference and direction for further development of DCE in the future.

**Method:** A bibliometric method was implemented using the Web of Science (WoS) Core Collection for the period from the database established to December 8, 2020. The data files are imported into CiteSpace and Excel to analyze and visualize the annual volume of productive, authors, countries, cited journals, cited articles, and keywords.

**Results:** A total of 1,811 articles were retrieved, then we read the abstract of each paper one by one, and 1,562 articles were included after screening, with an exponential increase in publication volume. John F. P. Bridges contributed to 40 publications and ranked first, followed by F. Reed Johnson (*n* = 37), Julie Ratcliffe (*n* = 36). The majority of the papers were conducted in the United States (*n* = 513) and the United Kingdom (*n* = 433). The top three cited journals were “*Health Economics*” (*n* = 981), “*Value in Health*” (*n* = 893), and “*Pharmaceutical Economics*” (*n* = 774), and the top three articles were “Constructing experimental designs for discrete-choice experiments: report of the ISPOR Conjoint Analysis Experimental Design Good Research Practices Task Force,” “Conjoint analysis applications in health-a checklist: a report of the ISPOR Good Research Practices for Conjoint Analysis Task Force,” and “Discrete choice experiments in health economics: a review of the literature.” The research hotspots and trends included “health technology assessment,” “survival,” “preference based measure,” and “health state valuation.”

**Conclusion:** The size of the literature about DCE studies in health care showed a noticeable increase in the past decade. The application of DCE in health care remains in an early growth phase, and “health technology assessment,” “survival,” “preference based measure,” and “health state valuation” reflected the latest research hotpots and future trends.

## Introduction

Discrete choice experiment (DCE), which grew primarily in Australia in the late 1970s, was pioneered by Louviere and Woodworth and introduced into the field of health economics by Propper (1). Discrete choice experiment was defined as a kind of preference elicitation technique asking respondents to make choice from two or more alternatives where at least one attribute is systematically varied and a series of choice tasks can elicit preferences (2, 3). Discrete choice experiment is based on three theories, namely, random utility theory (RUT), Lancaster's characteristics theory of demand, and the standard microeconomic theory of consumer (4). In recent years, DCE has gradually become the most commonly used method to quantify patients' health preferences (5), medical workers' employment preferences (6–8), and other stakeholders' preferences in health care, so as to provide decision-making basis for policy makers. The design and implementation of DCE includes the following steps: conceptualizing the choice process, selecting attributes and levels, experimental design, questionnaire design, pilot testing, sampling and sample size, data collection, coding of data, econometric analysis, validity, interpretation, and welfare and policy analysis (9).

Discrete choice experiment is one kind of method of stated preferences (10) that includes cardinal methods (time trade-off, standard gamble, and visual analog scale) and ordinal methods (DCE, ranking exercises, and ordered categorical responses) (3). As an ordinal method, DCE can reduce the cognitive burden of the responders, the complexity of the survey, and the measurement error compared with the cardinal method (3). Compared with other ordinal methods, such as ranking exercises, DCE shows the advantage of simulating a real-world situation where medical treatment or nursing attributes do not appear in isolation and being closer to the choice of patients (11). However, there are also deficiencies, such as the external effectiveness of DCE research that is very complex and difficult to measure, because the real data of personal health decision-making is usually not available. Researchers compare the advantages and disadvantages of different stated preference methods to provide the basis for other researchers to choose tools when conducting research (2, 12). Potoglou et al. (12) compared DCE with best–worst scaling (BWS), a sort of ranking exercises. The findings showed that DCE and BWS had no significant difference in assessing patients' preference weights, but BWS had less cognitive burden and provided more information than DCE. Skedgel et al. (2) compared DCE with constant-sum paired comparisons (CSPCs); DCE's completion rate and preference consistency are higher, but CSCP offers more advantages in eliciting preference for resources and/or outcomes distribution and attribute levels.

In order to better understand the research hotspots and development trends of DCE in health care, this study used bibliometric method to analyze the annual volume of productive, authors, countries, cited journals, cited articles, and keywords of DCE in health care. Bibliometrics is a statistical method, which can be used to study research trends, authors, publications, countries, and other related information in specific fields (13). This study used CiteSpace software based on Java environment developed by Professor Chaomei Chen, which has been widely used in various fields to analyze hotspots and trends (14). At present, there are few reports on the bibliometric and visual analysis of DCE studies in health care at home and abroad, so this study mainly uses this method to examine the application status of DCE in health care, so as to clarify its research trends.

## Methods

### Data Sources

An academic search of the Web of Science (WoS) core database was performed when it was established until December 8, 2020, using the following search strategy: TS = [ “discrete choice” AND (health * OR * care * OR medic * OR nurs * OR patient)]. The types of publications were limited to “articles” and only English papers. The inclusion criteria are as follows: (1) literature related to DCE studies; (2) literature related to medicine, nursing, pharmacology, psychology, neuroscience, health professions, and other healthcare fields. However, the search strategy might retrieve irrelevant results, for which the exclusion criteria are implemented: literature not related to health care and other fields related.

### Statistical Analysis and Ethics

The selected records were exported as plain text, and with the help of the analysis function of WoS, the quantitative statistics of literature publishing year and other information is carried out. CiteSpace 5.7 R3 (15) and Excel 2010 software were used to visually analyze the annual volume of productive, authors, countries, journals, and keywords of DCE in health care and draw relevant charts. Because this study does not involve people, there is no need for ethical approval.

## Results

### The Annual Volume of Productive

**Figure 1:**
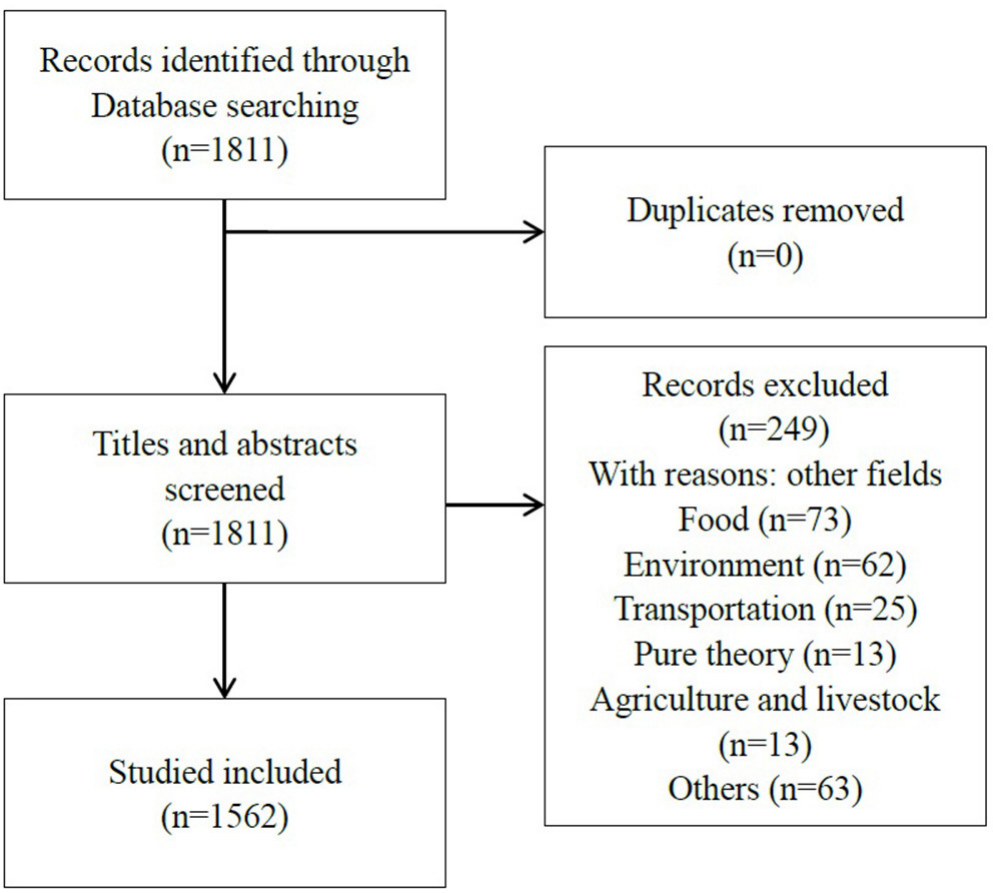
Flow diagram of study selection.

**Figure 2:**
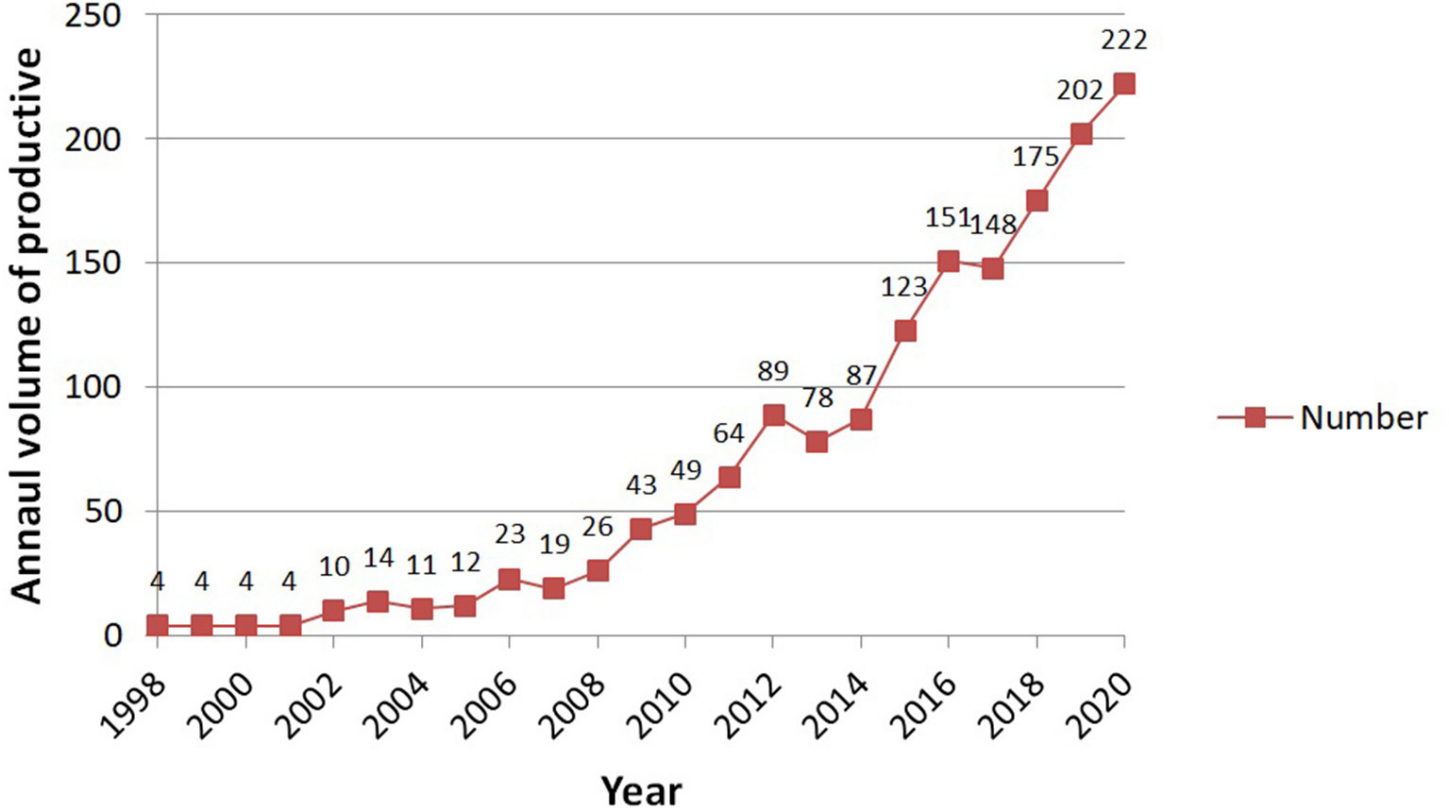
The annual volume of publication.

A total of 1,811 articles were retrieved; then, each paper's title and abstract was read one by one, and finally, 1,562 articles met the inclusion criteria (see Figure 1), with an average of 67.9 articles per year, and the volume of publications showed an obvious increasing trend. The annual volume of productive is shown in Figure 2. We identified a near exponential increase in the application of DCE in health care, with a slow increase from 1998 to 2005 and a rapid increase after 2005, with the largest increase in 2014–2015, illustrating the increasing use of DCE in health care.

### Authors

**Figure 3:**
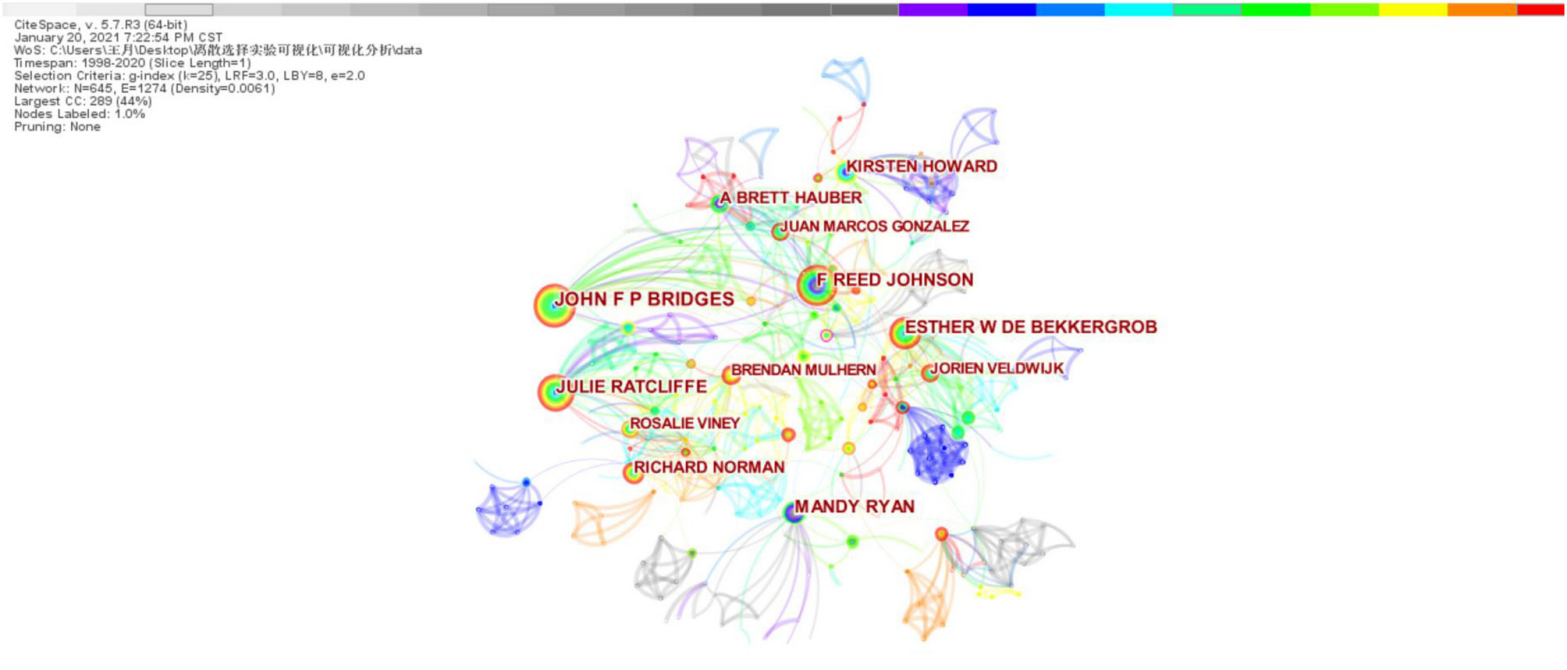
Author collaboration network.

As shown in Figure 3, the node size indicates the amount of papers published by the author. The larger the node size is, the higher the amount of papers published is. Among them, John F. P. Bridges has the largest number of nodes, which means that the author has published the most papers. The lines between different nodes indicate the cooperation, and the thicker the line is, the closer the cooperation is. Different colors indicate the time when the author published the papers. From the figure, it can be seen that each author has published more papers and cooperated more after 2011. In Figure 3, the number of network nodes is 645, the number of connections between nodes is 1,274, and the network density is 0.0061, which indicate that the cooperation between authors in this field is close and the academic atmosphere is strong.

The top 10 authors publishing DCE studies in health care are presented in Table 1. John F. P. Bridges (16), F. Reed Johnson (17), and Julie Ratcliffe (18) published the most articles, all more than 35. While Esther W. De Bekkergrob and Mandy Ryan published no more than 30 papers, their centrality (0.05 and 0.05, respectively) was greater than that of Julie Ratcliffe (0.04).

**Table 1:** The top 10 authors.

**Number**	**Author**	**Year**	**Count**	**Centrality**
1	John F. P. Bridges	2011	40	0.07
2	F. Reed Johnson	2007	37	0.06
3	Julie Ratcliffe	2009	36	0.04
4	Esther W. De Bekkergrob	2010	30	0.05
5	Mandy Ryan	2006	29	0.05
6	A. Brett Hauber	2009	26	0.02
7	Kirsten Howard	2011	24	0.04
8	Richard Norman	2014	24	0.01
9	Brendan Mulhern	2014	20	0.04
10	Jorien Veldwijk	2014	19	0.03

### Countries

**Figure 4:**
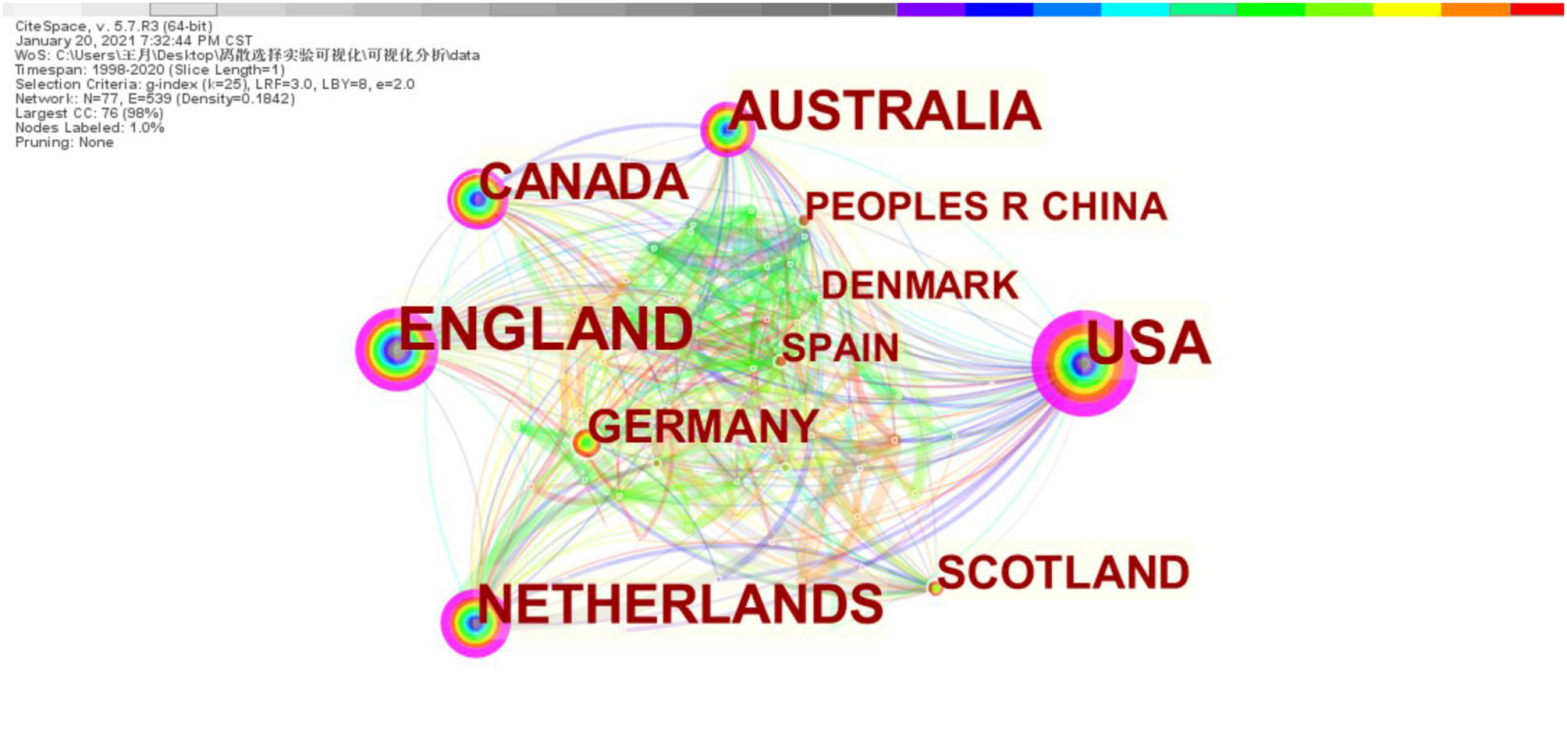
Countries' collaboration network.

As shown in Figure 4, different nodes represent different countries, with the node size reflecting the number of publications. The United States was the most dominant country according to the number of published papers. The thicker purple outer ring of the node represents the greater centrality, which means the stronger correlation with other nodes and the important influence. Lines between different nodes show cooperation between countries; different colors indicate the year the paper was published. In Figure 4, the number of network nodes is 77, the number of connections between nodes is 539, and the network density is 0.1842. This indicates that the cooperation between countries is very close, and a relatively stable cooperation network has been formed.

The top 10 countries publishing DCE studies in health care are presented in Table 2. The number of papers published in the United States and the United Kingdom is more than 400, and the centrality is large, indicating that the number, quality, and influence of articles written by authors in these two countries are large. It was followed by Australia (*n* = 291), the Netherlands (*n* = 255), and Canada (*n* = 180), with centrality ≥0.1. Although Germany published only 111 articles, its centrality was 0.1, indicating that the publication quality of the papers was good.

**Table 2:** The top 10 countries publishing discrete choice experiment (DCE) studies in health care.

**Number**	**Country**	**Count**	**Centrality**
1	USA	513	0.45
2	England	433	0.32
3	Australia	291	0.1
4	Netherlands	255	0.21
5	Canada	180	0.11
6	Scotland	115	0.05
7	Germany	111	0.1
8	People's Republic of China	53	0.01
9	Denmark	50	0.02
10	Spain	48	0.08

### Top Cited Journals

The top 10 cited journals are presented in Table 3. Among these 10 categories of journals, the top three journals with more than 350 citations were *Health Economics, Value in Health, Pharmacoeconomics* with an impact factor of 2.250, 4.748, 3.563, respectively, in which the impact factors of *BMJ-British Medical Journal, New England Journal of Medicine*, and *Lancet* were all above 30 (IF = 30.313, 74.699, 60.390, respectively).

**Table 3:** The top 10 cited journals.

**Number**	**Journal**	**Cited Frequency**	**Centrality**	IF^*^ 2019
1	Health Economics	981	0.01	2.250
2	Value in Health	893	0.01	4.748
3	Pharmacoeconomics	774	0.05	3.563
4	Social Science and Medicine	583	0.01	3.616
5	BMJ-British Medical Journal	517	0.02	30.313
6	Journal of Health Economics	512	0.01	2.827
7	Patient-Patient Centered Outcomes Research	466	0.01	3.226
8	Medical Decision Making	407	0.01	2.309
9	New England Journal of Medicine	356	0.02	74.699
10	Lancet	350	0.01	60.390

* *IF, impact factor*.

### Top Cited Articles

The top 10 cited articles in the field of DCE studies in health care are shown in Table 4. The article that received the highest citation, “Constructing experimental designs for discrete-choice experiments: report of the ISPOR Conjoint Analysis Experimental Design Good Research Practices Task Force,” was published in *Value in Health* in 2013 and received a total of 322 citations. All of the 10 cited articles are reviews or reports or guides or book, which summarize the application status of DCE in health economics and how to apply DCE in health care. The years of publication are 2008 and later. Besides “Using Discrete Choice Experiments to Value Health and Health Care” published in *The Economics of Non-Market Goods and Resources*, a non-medical journal, other articles are published in medical- and health-related journals.

**Table 4:** Top 10 cited references in discrete choice experiment (DCE) studies in health care.

**Number**	**Title**	**Year**	**Count**	**Centrality**	**Journal name**
1	Constructing experimental designs for discrete-choice experiments: report of the ISPOR Conjoint Analysis Experimental Design Good Research Practices Task Force (19)	2013	322	0.01	*Value in Health*
2	Conjoint analysis applications in health—a checklist: a report of the ISPOR Good Research Practices for Conjoint Analysis Task Force (20)	2011	311	0.02	*Value in Health*
3	Discrete choice experiments in health economics: a review of the literature (21)	2012	296	0.01	*Health Economic*s
4	Discrete choice experiments in health economics: a review of the literature (22)	2014	178	0.01	*Pharmacoeconomics*
5	Conducting discrete choice experiments to inform healthcare decision-making: a user's guide (9)	2008	173	0.02	*Pharmacoeconomics*
6	Statistical Methods for the Analysis of Discrete Choice Experiments: A Report of the ISPOR Conjoint Analysis Good Research Practices Task Force (23)	2016	161	0.01	*Value in Health*
7	Using qualitative methods for attribute development for discrete choice experiments: issues and recommendations (24)	2012	114	0.05	*Health Economics*
8	Sample Size Requirements for Discrete-Choice Experiments in Healthcare: A Practical Guide (25)	2015	113	0.01	*Patient–Patient Centered Outcomes Research*
9	Using Discrete Choice Experiments to Value Health and Health Care (26)	2008	96	0.01	*The Economics of Non-Market Goods and Resources*
10	Conjoint Analysis Applications in Health—How Are Studies Being Designed and Reported?: An Update on Current Practice in the Published Literature Between 2005 and 2008 (27)	2010	89	0.04	*Patient–Patient Centered Outcomes Research*

### Keywords

The burst strength of keywords indicates specific field hotspots and the future development trends. The top 20 keywords with the strongest burst strength in DEC studies in health care are shown in Table 5. These keywords cover many aspects, including joint analysis, willing to pay (WTP), value set, survival, health technology assessment, health care, preference-based measure, women's preference, etc. It is obvious that these keywords with the strongest burst strength can be divided into two broad categories. One is about DCE including conjoint analysis, WTP, value set, choice, and health technology assessment; the other is related with health including survival, symptom, health care, women's preference, and multiple sclerosis, which is consistent with the retrieval strategy.

**Table 5:** Top 20 keywords with the strongest burst strength publishing discrete choice experiment (DCE) studies in health care.

**Number**	**Keyword**	**Strength**
1	Conjoint analysis	12.35
2	Willing to pay	10.79
3	Task force	8.16
4	Practices task force	6.83
5	Value set	6.79
6	Contingent valuation	6.57
7	Conjoint analysis application	5.33
8	Choice	5.31
9	General practice	4.87
10	Survival	4.85
11	Perspective	4.72
12	Health technology assessment	4.68
13	Symptom	4.47
14	Strategy	4.39
15	Health care	4.3
16	Preference-based measure	4.2
17	Multiple sclerosis	3.83
18	Double blind	3.74
19	Women's preference	3.73
20	Health state valuation	3.72

**Figure 5:**
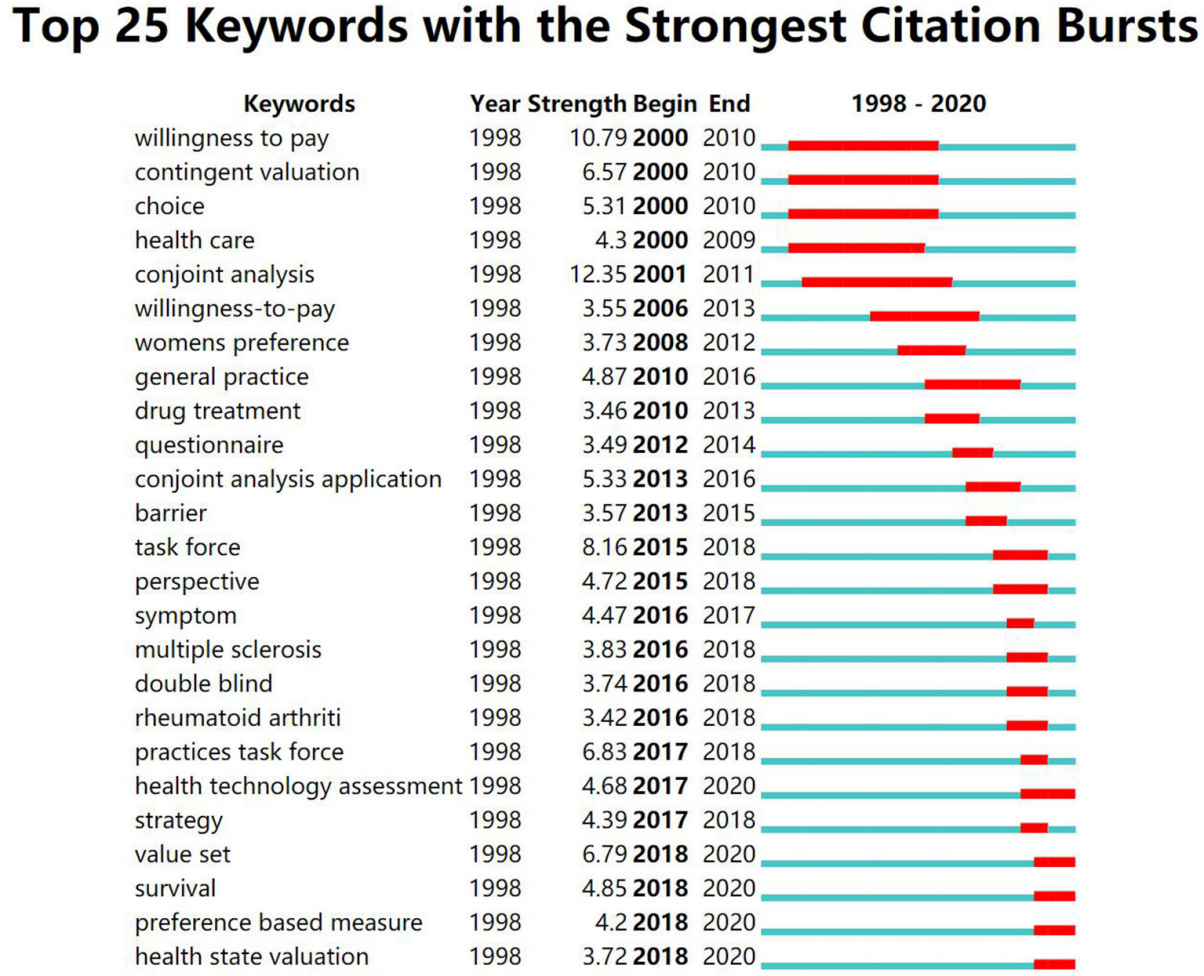
The citation burst of discrete choice experiment (DCE) studies in health care.

As shown in Figure 5, the line on the right of the figure indicates the time, and the red part indicates the active time of the keyword that was the research hotspot at that time. For example, the prominent time of WTP was from 2000 to 2010, and the research on WTP was the hotspot in the field in these 10 years. We also need to pay attention to the current research status and future research direction. As can be seen from the figure, health technology assessment, value set, survival, preference-based measure, and health state valuation are the research hotspots in DCE studies in health care at the present stage and may also be the research direction in the next few years.

## Discussion

### Summary

There are more and more researches on DCE in eliciting patients' preferences and health providers' employment preferences. The reasons are that patients' preferences play a key role in guiding treatment decision-making, especially in the absence of evidence-based decision rules, providing information for policy decision-making and affecting the treatment results (28). In this era of great pressure on medical staff and increasingly fierce contradictions between medical staff and patients, they have poor work enthusiasm and high turnover rate, which has become the primary task of healthcare system policy makers. Discrete choice experiment was used to evaluate the employment preference of medical staff for recruiting and retaining health workforce (29). Discrete choice experiment studies evaluating preference is becoming a trend.

From 1998 to 2020, the number of papers published in this field shows an exponential growth, which indicates that DCE has been paid more and more attention by experts and scholars in health care, and more and more research has been carried out. Among them, John F. P. bridges published the most papers, and he studies primarily joint studies application in health and patient preference. In terms of national publication volume, most of the articles with high publication volume and quality are concentrated in developed countries; especially the United States and the United Kingdom are in an absolute leading position, probably because their medical technology and philosophy are relatively advanced. At the same time, close cooperation is observed between different countries, which facilitates the sharing of technical knowledge and promotes the development of the field of medical and health. The top 10 cited journals were *Health Economics* (IF2019 = 2.250), the scope of which includes the determinants of health and its definition and valuation, as well as the demand for and supply of health care, planning and market mechanisms, microeconomic evaluation of individual procedures and treatments, and evaluation of the performance of health care systems (30). The majority of the journals belonged to the medical category, with the exception of *Social Science and Medicine* (IF2019 = 3.616), which belonged to social science, and *Patient Centered Outcomes Research* (IF2019 = 3.226), which belonged to nursing, but both were medically relevant.

### Knowledge Base

Co-citations can reflect the knowledge base of a field (31). In this study, the 10 most frequently cited articles in this field are listed, among which 4 are reviews, 3 are reports, 2 are guides, and 1 is a book, published in the top 10 cited journals. Almost all of them fall under the category of methodology research and reviews, aiming to provide guidance and offer the studies status quo for researchers or scholars interested in DCE research to apply DCE in health care.

The top 10 cited articles provide researchers with the application steps of DCE in the field of health and ISPOR practices task force had developed a checklist helping researchers to evaluate the integrity and rigor of their DCE studies (9, 20). More and more attention has been paid to the experimental design, a crucial step in DCE implementation. However, due to the lack of understanding of technology and software and the lack of consensus on the establishment of standards, the task force established by ISPOR is dedicated to summarizing and comparing many available methods for constructing experimental designs (19). Attribute generation methods for DCE include literature review, existing health outcome measures, professional recommendations, focus groups, interviews, patient surveys, and expert review, and qualitative work is very important for the generation of DCE attributes (including interviews, focus groups, and meta-ethnography), better reflecting the authenticity, and comprehensibility of attributes (24). The use of sample size calculations for DCE studies in health care is largely lacking, and the common methods often used are the rule of thumb and parametric method (25). There are multiple methods for analyzing data generated from DCE studies, and it is crucial to understand the characteristics and appropriate use of different analysis methods so as to conduct a well-designed DCE study (23).

These 10 articles mainly focus on the implementation methods of DCE, which are the bases. Every step in DCE study is constantly developing. It is suggested that a standard or specification of every step in the future research should be formed.

### Research Hotpots and Trends

The strong burst strength of keywords indicates the research hotspots and frontiers in a specific period of time and in a specific field (32). The top five strongest burst strengths of this study are conjoint analysis, WTP, task force, practices task force, and value set. Among them, conjoint analysis (2001–2011) contains DCE and other multi-attribute stated-preference methods, which is one method of eliciting preferences from stakeholders, especially patients (20). Value set (2018–2020) is the set of data generated by DCE, and WTP (2000–2010), as one of the outcomes of DCE, was a research hotspot during 2000–2010, which was consistent with the research results of de Bekker-Grob et al. (21). Task force (2015–2018) and practices task force (2017–2018) burst strength years in 2015–2018 because the lists and methods provided by the task forces established by ISPOR are widely used at this time.

Based on the burst strength and year, the following development trends of DCE studies in health care are listed:

(1) Assistance in Development Health Technology Policies

Health technology assessment (HTA) is a multidisciplinary process that summarizes information on medical, social, economic, and ethical issues related to the use of health technology in a systematic, transparent, fair, and robust manner (33). Researchers from various countries have reviewed the status of the national HTA project (17, 18, 34–38). The main function of the HTA project is to assist in the development of safe and effective health technology policies centered on patients (35). As regulatory and HTA decisions become more patient centric, approaches to measure the preferences of healthcare stakeholders are being explored, and DCE, as a tool that can effectively measure the preferences of patients and stakeholders, has been approved as a prescribed preference measure by regulatory and HTA agencies (16). Thus, how to apply DCE to accurately assess patients' preferences so as to assist in the development of safe and effective health technology policies centered on patients will become the trend of research in health care.

(2) Trade-Off Between Survival Rate and Other Factors in Cancer Patients

Discrete choice experiment is becoming more and more popular in the field of medical and health research, such as oncology, and can truly reflect clinical decision-making (39). The incidence of cancer accounts for 56.97% of the global incidence (40). There is a serious imbalance between the safety and effectiveness of the treatment of cancer patients. Therefore, the choice of treatment scheme is very important. Among the studies that elicit the preference of cancer patients, survival is the most commonly adopted attribute. Sugitani et al. (41) conducted a systematic literature review of quantitative patient preference studies of patients with lung cancer. The results showed that lung cancer patients tended to attach more importance to curative effect and quality of life (QoL) attributes, and overall survival rate was the most important of curative effect attributes, which was consistent with the results of Khan et al. (42) and Havrilesky et al. (43) using DCE to evaluate the preferences of patients with Hodgkin's lymphoma and ovarian cancer. However, Srinivas et al. (44) and Wong et al. (45) believed that it is more important for cancer patients to avoid adverse reactions when weighing survival rate and adverse reactions. Thus, it is suggested that in the treatment of different cancer patients in the future, we should first understand the trade-off between survival rate and adverse reactions, so as to provide decision-making basis for medical staff to make treatment plans.

(3) Comparing Different Preference-Based Methods for Health State Valuation

Preference-based methods can be classified into either cardinal methods or, more recently, ordinal methods, such as DCE (46). Bahrampour et al. (46) conducted a systematic review of DCEs to generate utility values for multiattribute utility instruments. Among the included studies, 24 studies used EQ-5D to evaluate the health status of patients through DCE, and there are also other multiattribute utility instruments, such as SF-6D, CHU-9D, and so on, using DCE to generate utility values. In addition to DCE, other methods such as SG and TTO are used to generate utility values for multiattribute utility instruments. Therefore, the study suggests that future studies can compare different preference-based methods for health state valuation. At present, DCE is still an important method to elicit preferences for valuing health states.

### Strengths and Limitations

In the past, there were some reviews describing the use of DCE in the field of health economics. For example, de Bekker-Grob et al. (21). conducted a systematic review of DCE health economics studies between 2001 and 2008, and Clark et al. (22) did that, too, but he focuses on the 2009–2012 literature. However, this study comprehensively analyzes the status and trends of DCE studies by using bibliometric method, not focusing on just health economics, but all related health care. Although this study was the first to analyze bibliometric indicators of DCE studies in health care, there were a few limitations. First, we only searched the WoS database, ignoring other databases, such as Scopus. However, this database collected a large number of high-quality articles and contains more than 12,500 magazines. Second, we limit the type of literature to articles in English, which may omit some other high-quality literature. Third, it takes a long time from the beginning of this study to the completion of the final paper, but according to the exponential growth trend of literature, a large number of literature will be published during the period of completion of the study, which is not included. Finally, there may be a potential bias that the same author may not use the same name when publishing in different magazines, or different authors may have the same name.

## Conclusion

Discrete choice experiment is a tool that can quantify preferences well. It has been applied more and more frequently in health care, and modern people are paying more and more attention to health status and pursuing better life quality, which provides a good opportunity for the development of DCE in health care. The application of DCE in health care should be strengthened in the future.

## Data Availability Statement

The original contributions generated for the study are included in the article/supplementary material, further inquiries can be directed to the corresponding author/s.

## Author Contributions

YW: designing this study and writing initial draft and revision. ZhangW and ZhaoW: reviewing the literature and analyzing. XL: making figures and tables. XP: rechecking the manuscript and putting forward suggestions for amendment. SW: revising language and content. All authors contributed to the article and approved the submitted version.

## Conflict of Interest

The authors declare that the research was conducted in the absence of any commercial or financial relationships that could be construed as a potential conflict of interest.
